# Maternal Creatine Supplementation Positively Affects Male Rat Hippocampal Synaptic Plasticity in Adult Offspring

**DOI:** 10.3390/nu11092014

**Published:** 2019-08-27

**Authors:** Stefano Sartini, Davide Lattanzi, Michael Di Palma, David Savelli, Silvia Eusebi, Piero Sestili, Riccardo Cuppini, Patrizia Ambrogini

**Affiliations:** Department of Biomolecular Sciences, University of Urbino Carlo Bo, I-61029 Urbino, Italy

**Keywords:** creatine supplementation, prenatal treatment, adult offspring, hippocampus, neuron excitability, long-term potentiation

## Abstract

Creatine plays a crucial role in developing the brain, so much that its genetic deficiency results in mental dysfunction and cognitive impairments. Moreover, creatine supplementation is currently under investigation as a preventive measure to protect the fetus against oxidative stress during difficult pregnancies. Although creatine use is considered safe, posing minimal risk to clinical health, we found an alteration in morpho-functional maturation of neurons when male rats were exposed to creatine loads during brain development. In particular, increased excitability and enhanced long-term potentiation (LTP) were observed in the hippocampal pyramidal neurons of weaning pups. Since these effects were observed a long time after creatine treatment had been terminated, long-lasting modifications persisting into adulthood were hypothesized. Such modifications were investigated in the present study using morphological, electrophysiological, and calcium imaging techniques applied to hippocampal Cornu Ammonis 1 (CA1) neurons of adult rats born from dams supplemented with creatine. When compared to age-matched controls, the treated adult offspring were found to retain enhanced neuron excitability and an improved LTP, the best-documented neuronal substrate for memory formation. While translating data from rats to humans does have limitations, our findings suggest that prenatal creatine supplementation could have positive effects on adult cognitive abilities.

## 1. Introduction

Creatine (N-(aminoiminomethyl)-N-methyl glycine) is a nitrogen-containing compound endogenously synthesized or exogenously obtained through diet, mainly meat or fish. It is a very popular ergogenic supplement for athletes but, recently, the use of creatine (Cr) supplementation has also been extended to the medical field.

In this regard, Cr supplementation has been successfully used as an adjuvant in the treatment of several myopathies, showing an ability to promote both muscle strength and increased lean mass in patients with dystrophies and polymyositis [1,2]. There is also evidence that in elderly sarcopenic patients, Cr supplementation in conjunction with resistance training protocols has beneficial effects on bone mass and strength [3], though a very recent meta-analysis study failed to show significant improvement [4].

In addition, patients with genetic creatine disorders (Arginine-glycine amidinotransferase (AGAT), Guanidinoacetate methyltransferase (GAMT), or Solute carrier family 6 member 8 (SLC6A8) deficiency) characterized by mental dysfunction were found to benefit from supplementation with Cr soon after birth [5], pointing to a possible association of creatine function with the cognitive performance [6]. However, male SLC6A8-deficient patients do not generally see a significant improvement in clinical markers following Cr supplementation [7]. Likewise, randomized clinical trials examining the cognitive effects of oral creatine supplementation in healthy individuals show that it may improve short-term memory and executive functions [8]. This evidence points to potential benefits of creatine supplementation for adult and aged healthy and diseased individuals. However, the ability of Cr to cross the blood–brain barrier in adulthood seems to be limited by the apparent absence of the SLC6A8 creatine transporter from astrocytes, mainly from their feet lining microcapillary endothelial cells [9], raising questions about the effectiveness of the treatment. On the contrary, during brain ontogenesis, Cr may be able to penetrate the blood-brain barrier more easily due to differences in the functioning of this brain interface [10]. This is of considerable importance, taking into account the role of creatine in brain development, function, and protection [11,12], and the limited capacity for creatine synthesis by the fetus. In fact, the fetus relies on the maternal creatine source, which is able to cross the human placenta [13]. Hence, creatine supplementation during gestation would result in increasing levels of creatine concentration in the developing brain. Indeed, thanks to its neuroprotective properties [11,12], Cr supplementation has been proposed as a preventive measure to protect the fetus during pregnancy [14] whenever oxidative stress arises, such as in cases of feto-placental hypoxia, fetal growth restriction, premature birth, mother malnutrition, or when parturition is delayed or complicated by oxygen deprivation of the newborn [15,16].

There is strong support for creatine use considering its safety profile and minimal risk for adverse events or the lack of any negative influence on markers of clinical health. However, possible morpho-functional effects of creatine supplementation on brain development have not been thoroughly investigated in vivo. We have recently demonstrated that maternal creatine supplementation (1% creatine in drinking water over the last ten days of pregnancy) increased pup cerebral creatine concentration of about 80% at postnatal 0 (P0), and affected the neural development of newborn rats [17]. In particular, we found that hippocampal CA1 neurons exposed to creatine loads during brain development showed a more extended and complex neurite tree together with larger synaptic responses, increased excitability, and enhanced long-term potentiation (LTP) [17]. Taken together, these findings indicate an alteration of the morpho-functional maturation of developing neurons under creatine supplementation conditions. It is noteworthy that the creatine was administered to dams from the 11th gestation day to the day before delivery, and the outcomes were investigated at the weaning age of pups, i.e., a long time after the termination of treatment. In addition, we previously evaluated hippocampus creatine content at the weaning age of pups (P21), and we found no difference between prenatally creatine-treated pups and age-matched controls (unpublished data). These findings suggest long-lasting modifications induced by prenatal creatine treatment that may persist into adulthood. If this were the case, an improved induction and maintenance of LTP would promote learning and memory processes, considering the role played by synaptic plasticity mechanisms in cognitive functions. On the other hand, enhanced neuron excitability could translate into higher network excitability resulting in an increased susceptibility to the generation of seizure episodes; however, our unpublished data do not show any spontaneous or induced epileptiform-like activity in hippocampal slices obtained from maternally creatine treated rat pups. Moreover, creatine supplementation is known to play a neuroprotective role in brain excitability by controlling the γ-aminobutyric acid (GABA)ergic function [18]. Therefore, in the present study, to verify possible long-term effects of prenatal creatine treatments we analyzed the morpho-functional features of hippocampal CA1 neurons in adult rats born from dams supplemented with creatine using morphological, electrophysiological and calcium imaging approaches.

## 2. Materials and Methods

### 2.1. Animals and Supplementation Protocol 

In this study, we used Sprague-Dawley albino rats (Charles River, Calco, Lecco, Italy). The use and care of the rats were conducted in accord with the guidelines of the Ethics Committee of the University of Urbino Carlo Bo (Prot. CESA 4/2011). Every effort was made to minimize the number of rats used. Virgin male and female rats (weighing 200–250 g) were housed in pairs with free access to food and water and maintained at an ambient temperature of 22 ± 1 °C with a 12-h light and 12-h dark cycle (lights on at 6 a.m. and off at 6 p.m.). After mating, female rats were randomly divided into two groups: 1. control group (control (CTRL); *n* = 4) drinking tap water; 2. supplemented group (treated (TREAT); *n* = 4) drinking tap water in which creatine (Fluka, Sigma-Aldrich, Milan, Italy) was dissolved (1 g/100 ml [19]) from the 11th day of pregnancy to the day before delivery (10 days of treatment). This supplementation period was chosen because of the expression of Cr membrane transporters at this time point [20]. The daily weight of the rats from the CTRL and TREAT groups was monitored throughout the experimental period, and no difference in the growth curves was found between the two groups. CTRL and TREAT male offspring were killed at ages ranging from postnatal day 60 to 70. The experiments were carried out by investigators blinded to the treatment groups.

### 2.2. Electrophysiological Experiment Preparation

Electrophysiological recordings were taken from the CA1 region of brain slices of adult male rats from CTRL and TREAT litters, as previously described [21,22,23]. Briefly, rats were anesthetized with an intraperitoneal injection of ketamine (65 mg/kg body weight) and killed by decapitation. Brains were then immediately removed, and four-hundred-micrometer-thick brain slices were prepared. The slices were allowed to recover for at least one hour in an interface recovery apparatus and then were transferred to the recording chamber. Following an equilibration period, field potential, whole-cell patch-clamp recordings were carried out. An analysis of electrophysiological traces was performed offline using a WinWCP software (Strathclyde Electrophysiology Software, John Dempster, University of Strathclyde, Glasgow, UK) or a WinFluor software (Strathclyde Imaging Software V 3.8.7, John Dempster, University of Strathclyde, Glasgow, UK) for calcium imaging measurements.

### 2.3. Field Potential Recordings 

The effect of maternal creatine supplementation on adult synaptic plasticity was investigated by evaluating the ability to elicit LTP in the Schaffer collateral-CA1 pathway (CTRL = 5 male rats; TREAT = 5 male rats). Recording micropipettes and bipolar stimulating electrodes were prepared, filled with artificial cerebrospinal fluid (ACSF) [21], and placed in the stratum radiatum of CA1 with approximately 300 µm distance between them. The extracellular recordings were carried out in slices showing extracellular field excitatory postsynaptic potentials (fEPSPs) of at least 1 mV in amplitude. Test pulses at 30-s intervals were applied to elicit baseline responses. Schaffer collaterals were then stimulated using 3 stimulus patterns every 5 seconds (every pattern included 10 trains of 100 Hz applied for 0.1 s separated by an interval of 1.2 s). The fEPSP was then monitored by 40 min recordings, and the fEPSP slope (between 10% and 80% of max) was analyzed and used as measures of synaptic strength; values were normalized to the mean value obtained over the last 20 min of the baseline period and expressed as a percent of this baseline value [22]. Before the application of the LTP induction protocol, the input–output relationship between the fiber volley amplitude and fEPSP slope was constructed increasing the Schaffer collateral stimulation intensity (from 0 to 140 µA, steps of 20 µA). 

### 2.4. Patch-Clamp Recordings 

We investigated the effects of maternal creatine supplementation on the electrophysiological properties of CA1 pyramidal cells using patch-clamp recordings in whole-cell configuration (CTRL = 5 male rats; TREAT = 5 male rats). Recordings were carried out under visual guidance, as previously described [21,22,23]. Patch-clamp pipettes were filled with internal solution (pH = 7.2, 290 mOsm) containing (in millimolar) 126 potassium gluconate, 8 NaCl, 0.2 ethylene glycol-bis(β-aminoethyl ether)-N,N,N′,N′-tetraacetic acid (EGTA), 10 4-(2-hydroxyethyl)-1-piperazineethanesulfonic acid (HEPES), 3 Mg_2_ATP, 0.3 GTP, and biocytin (Sigma-Aldrich, Milan, Italy, 0.2%) for subsequent determination of cell morphology. The electrode resistance ranged from 3 to 5 MΩ. The junction potential between internal and external solutions was not corrected. Cells to be recorded were identified in the CA1 pyramidal cell layer based on their typical shape. Resting membrane potential (RMP), input resistance (IR), membrane capacitance (C), and cell excitability were determined as previously described [21]. The amplitude and shape of the first elicited action potential and the relationship between depolarizing injected current and percent of cells that elicit action potential were evaluated. The afterhyperpolarization (AHP) was triggered using a depolarizing current step of 1 s able to induce maximum firing rate. The bipolar stimulating electrode was filled with ACSF solution and placed in stratum radiatum approximately 300 µm from the recording cell. Postsynaptic potentials (PSPs) were recorded in current-clamp mode as previously described [21,22]. During PSP recordings, cells were held at a membrane potential of −70 mV. Paired stimuli with an interstimulus interval of 50 ms were delivered every 20 s to the Schaffer collaterals and the amplitude of first and second excitatory post synaptic potentials was measured. To evaluate the effect of prenatal creatine supplementation on resting membrane potential, we considered the RMP of all neurons and recordings were rejected only if the initial series resistance was >30 MΩ, if the series resistance measured at the end of the experiment had changed (±5 MΩ), or if DC offset exceeded 5 mV after withdrawal from the cell.

### 2.5. Morphological Analysis

After patch-clamp recordings, the slices were fixed with paraformaldehyde (4% PFA in PBS; Sigma–Aldrich, Milan, Italy) overnight at room temperature, and biocytin, injected by patch recording electrode, was revealed as previously described [21,22]. Morphological reconstruction of each labeled pyramidal cell was performed using a Leica TCS-SL confocal microscope (Leica Microsystems Srl, Milan, Italy), equipped with Argon and He/Ne laser sources. Neurons without clear dendritic cutting at the slice surface were considered for morphological analysis. The total length of the basal and apical dendritic trees of CA1 pyramidal cells was assessed using the image analysis software NeuronJ (version 1.4.3, ImageJ, Imagescience.org, Sydney, Australia). Furthermore, Sholl concentric ring analysis was applied to evaluate dendritic tree complexity, as previously described [22].

### 2.6. Calcium Imaging

Calcium imaging recordings in whole-cell configuration were performed (CTRL = 8 male rats; TREAT = 8 male rats), and the experiments were carried out under visual guidance using a Zeiss Axioskop microscope (Carl Zeiss International, Italy) equipped with a 40× water immersion objective and an Orca Flash 4.0 Charge-Coupled Device (CCD) camera (C11440, Hamamatsu, Japan).

All recordings were performed on CA1 pyramidal neurons using an Axopatch-200B amplifier (Axon Instruments, Foster City, CA, USA). Patch pipettes were filled with an intracellular solution containing in millimolars: 126 potassium gluconate, 8 NaCl, 10 HEPES, 3 Mg_2_ATP, 0.3 GTP (pH = 7.2; 290 mosM). To evaluate cellular calcium changes, Fluo-4 pentapotassium salt (100 µM) was added to the internal solution.

Fluorescence images (200 × 200 pixels) were acquired at 100 Hz frequency using a FITC excitation filter of 450 to 490 nm and fluorescence values were expressed as ΔF/F, where F is the fluorescence at resting condition: the region of interest (ROI) (10 × 10 pixels) was placed on the cell body, and the background fluorescence was sampled in a region far from the loaded cell. We minimized photobleaching by illuminating the slice with minimal light intensity. 

Fluorescence evaluation started immediately after break-in. During the loading phase, F_0_ and the calcium transient in response to a single action potential were monitored; the single action potential was evoked in current-clamp mode every 30 s using brief current pulses (2 ms), and the neurons were held at about −70 mV to avoid spontaneous action potentials. The steady-state was reached after about 15 min. Moreover, once cells were fully loaded with Fluo-4, fluorescence changes in response to high-frequency stimulations (20–100Hz, 600 ms) were evaluated.

In each neuron, unperturbed calcium transient, intracellular calcium concentration at resting condition [Ca^2+^]_0_ and during a single action potential Δ[Ca^2+^]_AP_ were calculated according to Maravall et al. [24]. Calcium concentration was obtained using Equation (1):

(1) $$\frac{\Delta \left[Ca^{2+}\right]}{K_{d}}=\frac{F_{max}}{F_{0}}\left(1-R_{f}{}^{-1}\right)\frac{\Delta F/F}{\left({\left(\Delta F/F\right)}_{max}-\Delta F/F\right)\cdot {\left(\Delta F/F\right)}_{max}},$$

where Δ[Ca^2+^] is the transient in calcium concentration, K_d_ is the Fluo-4 dissociation constant (345 nM), F_max_ is the fluorescence measured at saturation, F_0_ is the fluorescence during resting condition, R_f_ is the dynamic range of the indicator (we considered a value of 85 as reported for Fluo-4 in Maravall et al. [24]), ΔF/F is the rise in fluorescence divided by the resting fluorescence, (ΔF/F)_max_ is the ΔF/F at saturation. F_max_ was measured in every cell using high-frequency action potential trains (from 20 to 100 Hz) to obtain maximal ΔF/F values.

The calcium concentration at resting condition [Ca^2+^]_0_ was determined using Equation (2):

(2) $$\frac{{\left[Ca^{2+}\right]}_{0}}{K_{d}}=\frac{1-R_{f}{}^{-1}}{{\left(\Delta F/F\right)}_{max}}-R_{f}{}^{-1}.$$

The buffer capacity of the indicator (k_B_) was calculated using Equation (3):

(3) $$K_{B}=\;\frac{K_{d}\times {\left[B\right]}_{T}}{\left(K_{d}+{\left[Ca^{2+}\right]}_{0}\right)\times \left(K_{d}+{\left[Ca^{2+}\right]}_{peak}\right)},$$

where [B]_T_ is the Fluo-4 concentration (in nM) and [Ca^2+^]_peak_ is the calcium concentration at the peak of the calcium transient.

In both experimental groups, the endogenous buffer capacity (k_S_) was evaluated back-extrapolating the relationship between the reciprocal of calcium transient amplitude during a single action potential (1/Δ[Ca^2+^]_AP_) and the exogenous buffer capacity (k_B_). The data obtained were fitted with a straight line and the intercept of this line with the x-axis provide an estimation of endogenous buffer capacity (k_S_ = −(1 + x intercept)).

### 2.7. Statistical Analysis

Data were analyzed using Prism 7 (GraphPad Software, La Jolla CA, USA) or SPSS 25 (IBM Corp., Armonk, NY, USA) and expressed as Mean ± (standard error of mean) SEM. As dictated by data distribution, differences between the experimental groups were statistically evaluated by appropriately applying Student’s *t*-test or χ square or two-way ANOVA followed by Sidak’s post hoc test. The relationship between the fEPSP slope and fiber volley amplitude was evaluated using linear regression. For all analyses, the significance threshold was established at *p* = 0.05.

## 3. Results

### 3.1. Field Recordings

**Figure 1:**
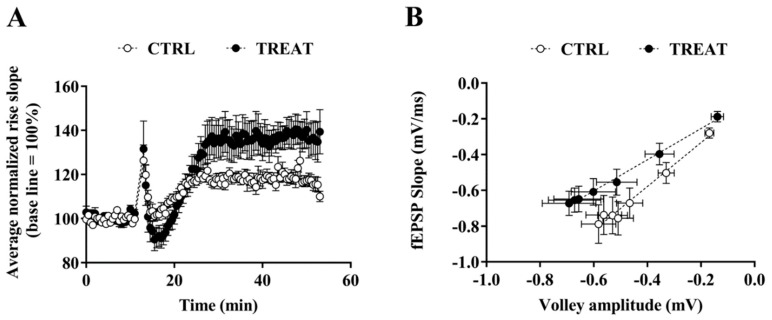
Effects of maternal creatine supplementation on basal synaptic transmission and long-term potentiation (LTP) in adult Cornu Ammonis 1 (CA1) neurons. (**A**) High-frequency stimulation of Schaffer collaterals results in a more pronounced LTP in treated CA1 neurons. The field excitatory postsynaptic potential (fEPSP) slope (between 10% and 80% of max) was analyzed as a measure of synaptic strength; values were normalized to the mean value obtained over the last 15 min of the baseline period and expressed as a percentage of this baseline value. Two-way ANOVA with *Time* and *Treatment* (CTRL vs TREAT) as independent variables while the *litter* was considered as a blocking factor; *Time*: F (1, 2692) = 861.69 *p* < 0.001, *Treatment*: F (1, 2692) = 103.24 *p* < 0.001, *Time-by-Treatment Interaction*: F (1, 2692) = 117.33 *p* < 0.001 (control, CTRL: *n* = 11 slices; treated, TREAT: *n* = 16 slices). (**B**) Input/output curves for treated and control rats were measured by plotting fEPSP slopes and their corresponding presynaptic fiber volley amplitudes at increasing stimulus intensities. Basal synaptic transmission is altered in treated (*n* = 17 slices) rats compared to controls (*n* = 18 slices). Linear regression *p* = 0.0011. CTRL = 5 rats; TREAT = 5 rats.

Exposure to Cr supplementation during brain development affected LTP in CA1 neurons in adult rats: high-frequency stimulation (HFS) of stratum radiatum induced an intense LTP that increased significantly over time compared to the control group (Figure 1A). This finding confirms the previous results obtained in immature rats [17] and shows that LTP modification persists in adulthood. This result is consistent with data depicted in Figure 1B, which show the relationship between the EPSP slope and the volley fiber amplitude. In particular, neurons from the creatine-exposed group exhibited a higher number of activated synapses with a lower on average EPSP than controls, indicating a less efficient synaptic transmission.

### 3.2. Whole-Cell Analysis

On average, CA1 neurons from Cr-supplemented and control rats did not appear to be significantly different in terms of membrane passive properties (IR and C), RMP, or action potential threshold (Table 1). 

**Figure 2:**
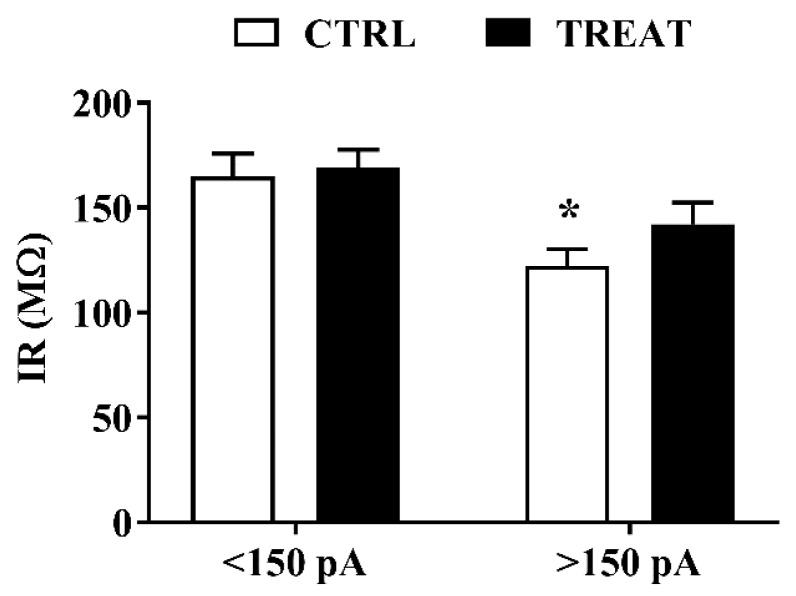
Creatine maternal supplementation affects input resistance in adult CA1 neurons. Neurons of treated rats excited by current injections above 150 pA do not show differences in input resistance compared to treated neurons excited by current injections below 150 pA, while such differences were found in neurons of control rats. Two-way ANOVA with *Intensity of stimulation* and *Treatment* (CTRL vs TREAT) as independent variables while the *litter* was considered as a blocking factor; *Intensity*: F (1, 42) = 14.69 *p* = 0.0004; >150 pA CTRL: *n*=11 neurons; TREAT: *n* = 11 neurons; <150 pA CTRL: *n* = 14 neurons; TREAT: *n* = 11 neurons. Sidak’s multiple comparisons test: <150 pA CTRL vs. >150 pA CTRL * *p* = 0.0116. CTRL = 5 rats; TREAT = 5 rats.

However, neurons from the Cr-treated group exhibited higher excitability during step by step incremental stimulation current. Indeed, current injection at low intensity (200 pA) was able to elicit action potentials in 100% of the recorded Cr-exposed neurons, unlike control neurons, which required a higher current intensity (350 pA). As no difference in action potential threshold and membrane passive properties was pointed out, to provide an explanation to this finding, IR was reconsidered to identify possible individual differences among neurons within the two experimental groups. In fact, we discovered that neurons from controls which required high current intensity to evoke action potentials, showed a significantly lower IR making them less excitable (Figure 2).

**Figure 3:**
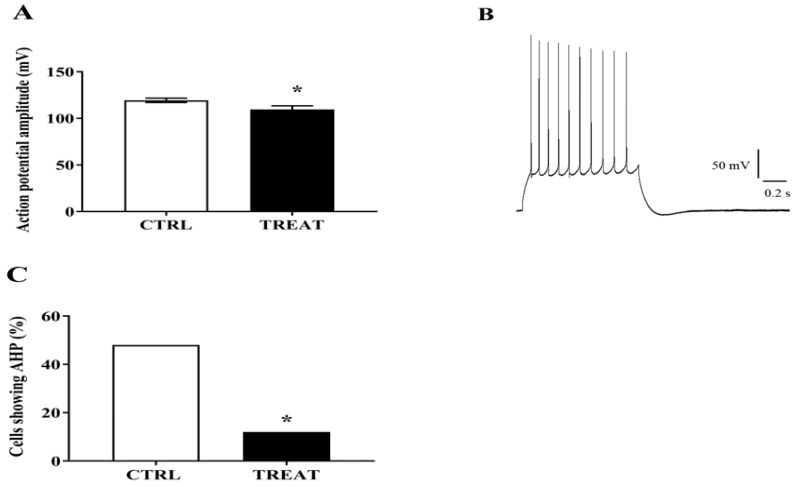
Creatine prenatal treatment effects on CA1 pyramidal cell excitability in adult progeny. (**A**) Neurons of treated rats show lower action potential amplitude than control neurons. Unpaired *t*-test * *p* = 0.033; CTRL = 25 neurons; TREAT = 25 neurons. (**B**) Current-clamp recording of action potentials generated by direct intracellular injections of depolarizing current (bringing the membrane potential from approximately −70 to −10mV). An afterhyperpolarization is observable following action potential burst. (**C**) Creatine supplementation reduces the afterhyperpolarization appearance in CA1 neurons. CTRL = 25 neurons; TREAT = 25 neurons. Fisher’s exact test: * *p* = 0.012. CTRL = 5 rats; TREAT = 5 rats.

Analyzing the amplitude of CA1 neuron action potential, we found that it was significantly lower in the creatine-treated group compared to controls. Moreover, only 12% of these neurons showed an afterhyperpolarization (AHP) following spike burst induction, in comparison to 48% detected in the control group (Figure 3A–C). This result would support the improvement in LTP maintenance obtained in Cr-exposed group (see below), as AHP was found to be inversely related to LTP induction facility [25]. 

Finally, when postsynaptic potentials were evoked by stimulating Schaffer collaterals, EPSP and inhibitory postsynaptic potential (IPSP) amplitudes did not show significant differences.

### 3.3. Morphological Analysis 

**Figure 4:**
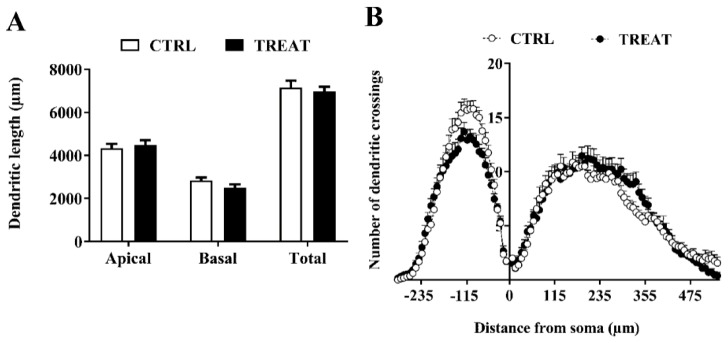
Morphological analysis of biocytin-stained neurons from the CA1 region of the hippocampus. Creatine maternal supplementation does not affect neuronal morphology in adult progeny. (**A**) Dendritic length of the CA1 pyramidal cells. (**B**) Scholl analysis plot showing numbers of dendritic crossings along the Scholl rings as a function of distance from the soma. Distributions of both basal and apical dendritic crossings do not result in significant differences between the two groups, two-way ANOVA with Distance from soma and Treatment (CTRL vs. TREAT) as independent variables while the litter was considered as a blocking factor; CTRL: *n* = 16 neurons; TREAT: *n* = 13 neurons. CTRL = 5 rats; TREAT = 5 rats.

To evaluate whether morphological modifications in hippocampal neurons previously observed in immature treated rats [17] persisted into adult life, we carried out measurements of CA1 neuron dendrite length and complexity from maternally Cr-supplemented 60-day-old rats (two months after the termination of creatine treatment). Morphometric differences were no longer recognizable in adulthood. Indeed, as shown in Figure 4, both dendrite length (A) and complexity (B) were very similar in the two experimental groups.

### 3.4. Intracellular Calcium Dynamics

**Figure 5:**
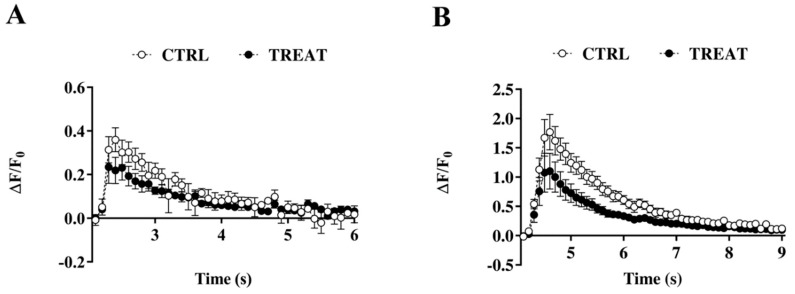
Intracellular somatic calcium transients. (**A**) Calcium transients evoked by single action potentials; (**B**) calcium transients evoked by 300 ms-20 Hz action potentials burst. Creatine maternal supplementation significantly decreases calcium influx during 20 Hz action potential burst in CA1 neurons. Two-way ANOVA with *Time* and *Treatment* (CTRL vs. TREAT) as independent variables while the *litter* was considered as a blocking factor; *Time*: F (1, 645) = 305.47 *p* < 0.001, *Treatment*: F (1, 645) = 51.74 *p* < 0.001, *Time-by-Treatment Interaction*: F (1, 645) = 19.02 *p* < 0.001. Sidak’s multiple comparisons test: *p* < 0.05 from 4.9 to 5.6 s. CTRL = 8 neurons from 5 rats; TREAT = 5 neurons from 4 rats.

No difference was found in resting Ca^2+^ concentrations between the treatment and control group (52.22 nM ± 7.75 vs. 41.25 nM ± 5.68) or in endogenous total buffering capability (Cr: 114.3; CTRL: 92.5). Likewise, [Ca^2+^]_I_ transients induced by evoking a single action potential were not significantly different in Cr-exposed rats compared to controls (Figure 5A). However, a trend showing lower [Ca^2+^]_I_ levels was observed in the Cr-treated group, which increased when neurons were stimulated to elicit a prolonged action potential firing (20 Hz, 300 ms) (Figure 5B), becoming significantly different from controls.

## 4. Discussion

In the present study, we investigated the long-term effects of maternal creatine supplementation on morphological and functional features of CA1 pyramidal neurons in adult offspring of rats. This work arose from a previous investigation where we demonstrated that creatine supplementation over the last 10 days of rat pregnancy induced a significant accumulation of creatine in pup brain [17], promoting rapid morphological and functional maturation of CA1 pyramidal neurons. These findings were consistent with the creatine effects that we found in neuroblast primary cultures [26]. In line with these findings, an enhanced LTP was also found [17] in supplemented newborn rats. 

The results of the present investigation build on our previous findings, revealing that adult offspring of dams exposed to creatine during pregnancy retained some neuronal differences compared to age-matched controls, while other differences were no longer detectable. In particular, the extension and complexity of dendritic arbors became very similar to those of controls in adulthood. As pyramidal neurons in the hippocampal CA1 region are generated in the Ammonic neuroepithelium mainly from E16 to E20 in rats [27,28], we may speculate that creatine supplementation during this period of development speeds up neuronal maturation probably by providing more substrate for the transfer processes and by increasing cellular energy reserves in the form of PCr [29] to sustain cytoskeleton dynamics for dendritic development. 

From a functional point of view, it is well known that during postnatal brain development, passive membrane properties (IR and C) and RMP of CA1 pyramidal neurons changes; in details, IR decreases, C increases, and RMP becomes less depolarized [30]. In our previous work [17], we found that CA1 neurons of TREAT pups showed a normal maturation of passive properties, but a more depolarized RMP compared to CTRLs. This difference disappeared in TREAT adult rats. Two-months after the end of creatine exposure, the difference in spike threshold, which was close to RMP values in treated pups and significantly different from controls, was no longer observable. This suggests that a massive inactivation of voltage-gated ion channels may have occurred in controls but not in treated pups. This difference did not appear to persist in two-month-old rats. Overall, these findings led us to hypothesize that changes in ATP availability might have affected ion channel phosphorylation, altering their kinetics, and thus changing the electrical properties of neuronal cell membranes under creatine exposure. 

On the other hand, the current intensity capable of eliciting action potentials in CA1 neurons was much lower in maternally Cr-exposed rats, highlighting an enhanced excitability, which appears to be closely related to neuron input resistance. This points to the existence of some difference in passive ion channel expression and/or in membrane translocation able to increase membrane resistance and thus the excitability of these neurons. 

Neuronal excitability regulation is also related to AHP [31]. Intracellular Ca^2+^ transients, generated by the activity of L-type channels and calcium release from stores [31,32,33], have been shown to play a role in AHP modulation [25]. In particular, calcium release from intracellular stores was found to affect AHP shape [32] and to contribute to AHP enhancement during aging [33,34,35]. In this scenario, our current findings showed that most adult CA1 neurons prenatally treated with creatine did not exhibit AHP following action potential burst and showed lower [Ca^2+^]_I_ levels, mainly when they were stimulated to elicit a prolonged action potential firing. Since the post burst AHP is predominantly a Ca^2+^-dependent K^+^ current [25,36], it can be hypothesized that in neurons prenatally exposed to Cr supplementation, a lower evoked calcium transient may result in a minor activation of calcium-dependent potassium channels and consequently in a reduction of AHP expression.

Interestingly, numerous studies show that AHP amplitude is related to LTP induction [25,37,38,39,40] with a relatively large AHP that was found to underlie LTP impairment in aged animals [41,42]. In line with these findings, we observed an enhanced LTP in CA1 neurons of the Cr treated group. However, the intense LTP recorded in this group of rats may also be consistent with our data showing a higher number of activated synapses with lower average stimulus intensity and greater intrinsic excitability than controls (see Figure 1B and Figure 2). Finally, these functional features can allow a higher level of associativity and cooperativity, conditions that typically trigger the LTP in CA3–CA1 synapses [43]. In any case, it is remarkable that LTP is a major reflection of synaptic plasticity, in addition to being the best-documented neuronal substrate for memory formation [44,45,46], thus suggesting a potential beneficial impact of maternal Cr supplementation on learning processes in adulthood. The impairment of hippocampus-dependent learning ability in adult mice in which the SLC6A8 gene of the Cr transporter is knocked out at P5, but not when the gene is deleted at P60 [47], points to the role of Cr in hippocampus-related cognitive function development.

Taken together, our findings point to the possible modulation of ion channel expression and/or kinetic by Cr supplementation which occurs during fetal life, and persists, in part, into the adulthood. In addition to its predominant role in energy homeostasis, Cr has been reported to exert several actions independent of ATP balancing [11]. Indeed, in vitro and in vivo studies report the ability of Cr to modulate neurotransmitter receptors and ion channels. For example, the activity of brain GABA_A_ receptor is increased by i.c.v. Cr injection [48] and, moreover, Cr is known to interact with the benzodiazepine site of these receptors [16]. Furthermore, activation of adenosine A1 and A2 receptors by Cr produces antidepressant effects [49]. Likewise, Cr supplementation in vitro has been shown to increase Na^+^ and K^+^ currents in spinal neuroblasts [26], possibly as a consequence of enhanced voltage-gated ion channel expression or membrane translocation, and to accelerate the expression of the mature form of voltage-gated ion channels in rat CA1 neurons [17]. In this context, it is worth noting that neuron gene expression can be influenced by Cr via brain-derived neurotrophic factor (BDNF) modulation [50] and that BDNF signaling has been shown to regulate ion channel membrane trafficking [51]. 

In short, our previous and current results show that maternal supplementation with creatine can affect morpho-functional maturation of neurons in developing hippocampus and promotes permanent effects on synaptic plasticity in adult offspring, although future studies are needed to address the mechanisms by which Cr mediates its effects. In principle, extrapolation from rats to humans is questionable and not free from bias; thus, our findings cannot be directly transferred to humans. However, we demonstrated that even very high doses of creatine do not induce negative effects on rat hippocampal neurons. On the contrary, our results suggest that prenatal creatine supplementation may have positive effects of on adult cognitive abilities.

## Figures and Tables

**Table 1:** Electrophysiological features. Analysis of Cornu Ammonis (CA1) pyramidal neurons in adult offspring from creatine-supplemented and unsupplemented mothers.

	*n*	RMP (mV)	IR (Mohm)	C (pF)	AP Threshold (mV)	EPSP Peak (mV)
CTRL	25	−67.0 ± 2.9	140.9 ± 7.8	183.4 ± 10.4	−54.0 ± 1.5	9.3 ± 1.3
TREAT	28	−63.1 ± 2.9	137.9 ± 8.8	171.3 ± 9.0	−55.6 ± 1.3	6.4 ± 0.9
